# Factors affecting postoperative mortality of patients with insufficient union following osteoporotic vertebral fractures and impact of preoperative serum albumin on mortality

**DOI:** 10.1186/s12891-020-03564-z

**Published:** 2020-08-10

**Authors:** Tetsuro Ohba, Hiroshi Yokomichi, Kensuke Koyama, Nobuki Tanaka, Kotaro Oda, Hirotaka Haro

**Affiliations:** 1 Department of Orthopaedic Surgery, University of Yamanashi, 1110 Shimokato, Chuo, Yamanashi, 409-3898 Japan; 2 Department of Health Sciences, University of Yamanashi, Yamanashi, Japan

**Keywords:** Osteoporotic vertebral fractures, Mortality, Serum albumin

## Abstract

**Background:**

Numerous comparative studies of surgical procedures have focused on clinical and radiographical outcomes, as well as the effect of bone fragility on the outcome of spinal surgery; however, insights concerning a risk of mortality or morbidity have been limited. Additionally, the effect of surgical therapy on survival after vertebral compression fractures remains controversial. Our aim was to evaluate the preoperative factors that affected the long-term survival of patients who underwent spinal surgery for an insufficient union following osteoporotic vertebral fractures (OVF) and to determine postoperative mortality.

**Methods:**

We retrospectively reviewed the cases of 105 consecutive patients who underwent spinal surgery for OVF. Mortality was estimated using the Kaplan-Meier method and a log-rank test. The preoperative backgrounds of patients were analyzed to determine which risk factors led to death among the OVF cases. Kaplan-Meier curves were used to estimate survival based on preoperative albumin levels of ≤3.5 g/dL (hypoalbuminemia) versus > 3.5 mg/dL.

**Results:**

The mean follow-up time was 4.1 ± 0.8 years. Two years after surgery, percentage of patients who had died was 15%. The VAS scores and modified Frankel classification were significantly improved one year after surgery. The ratio of male-to-female was significantly higher for patients with OVF who died than for those who were still alive. No significant difference in mortality was observed among surgical procedures for OVF. The univariate analysis showed that male gender, serum albumin < 3.5 g/dl, creatinine clearance< 60 mg/dl, and the American Society of Anesthesiologists classificat0ion ≥3 were significant risk factors for postoperative mortality. Multivariate analysis revealed that only serum albumin ≤3.5 g/dL was a significant risk factor for long-term postoperative mortality of patients with OVF.

**Conclusions:**

Preoperative hypoalbuminemia was associated with postoperative mortality following surgery for OVF.

**Level of evidence:**

Level 3.

## Background

Osteoporotic vertebral fractures (OVF) are the most common type of fragility fractures, and they have become more prevalent as the proportion of the population that is older continues to increase [1]. Reports indicate that OVF increase overall mortality [2, 3]. Conventionally, OVF are viewed as benign and treated with conservative methods such as rest, immobilization, drugs, and bracing [4]. However, vertebral fractures sometimes fail to unite, resulting in progressive kyphosis due to vertebral collapse and/or pseudarthrosis. Affected patients often endure persistent back pain and/or neurological deficits [5]. Despite conservative treatment, the prevalence of an insufficient bone union among elderly patients with OVF reportedly ranges from 10.0 to 13.5%. The quality of life and activities of daily living of these patients may be reduced severely [6, 7]. Despite the various surgical procedures proposed for the management of an insufficient bone union following OVF, optimal surgical procedures have not yet been clearly established. This may be because of the poor general condition and frequent instrumentation failure resulting from low bone quality in elderly patients. To treat these patients, a less invasive surgical approach in combination with more rigid fixation may be optimal [8], but some patients still require revision surgery because of progression of their kyphotic deformity, instrumentation failure, or both. Numerous comparative studies of surgical procedures have focused on clinical and radiographical outcomes, as well as the effect of bone fragility on the outcome of spinal surgery [9, 10]. However, insights concerning the risk of postoperative mortality or morbidity are limited. Additionally, the effect of surgical therapy on survival after vertebral compression fractures remains controversial [11, 12]. To establish an effective treatment strategy for patients with OVF, the postoperative mortality of patients who have undergone surgery for OVF, and the preoperative risk factors predictive of mortality after surgery, should be elucidated.

In the present study, we sought to evaluate the preoperative factors affecting the postoperative mortality of patients who underwent spinal surgery for an insufficient bone union following OVF and determine postoperative mortality.

## Methods

### Patient cohort and surgery

This study was a retrospective review of prospectively collected data. A total of 105 consecutive patients, with delayed osteoporotic thoracolumbar vertebral fractures, who underwent spinal surgery were studied (Table 1). Surgeries were conducted by three board certified spinal surgeons at a single institution between 2010 and 2018. Initially, all patients with OVF were treated conservatively, and none of them had neurological deficits immediately after injury. Treatment options, including the use of a brace or drug therapy, or both, were selected by individual physicians based on their experience. Additionally, drug treatment for osteoporosis was started at the discretion of each physicians after visiting hospital. Surgical treatment was indicated for all 105 patients because they had progressive neurological deficits or continuous severe lower back pain caused by vertebral collapse. Indications for surgery in neurologically intact patients were persistent pain following 3 months of conservative treatment, presence of pseudoarthrosis & progressive disability & deformity.

**Table 1:** Preoperative characteristics and demographics of patients with OVF^a^

Patients with OVF	(*N* = 105)
Age at the time of admission, y	76.3 ± 7.5
Gender, female/male, n	65/40
BMI (kg/m^2^)	22.4 ± 3.3
BMD (%YAM)	69.7 ± 13.5
Follow-up duration, months	28.3 ± 31.8
Injured vertebral level (Th10-L2/L3–4)	77/28
Type of Denis’s classification (A/B/C)	56/46/3
**Intraoperative technique, n**	
VP (HA) + PF	49
BKP	18
ADF + PF	38

^a^Mean ± standard deviation, unless otherwise indicated

*ADF* anterior spinal decompression and fusion, *BKP* balloon kyphoplasty, *BMI* body mass index, *BMD* bone mineral density, *YAM* young adult mean, *HA* hydroxyapatite, *OVF* osteoporotic vertebral fractures, *PF* posterior fixation, *VP* vertebroplasty

Three surgical procedures were applied in the treatment of the patients in this study (Table 1). Forty-nine patients underwent posterior decompression and short-segment fixation with vertebroplasty at the fractured level (VP (HA) + PF). After reduction, a 5 or 6-mm-diameter hole was made into the pedicles of the fractured vertebra bilaterally with a pedicle probe. A cavity extending to the anterior part of the vertebra was then created via both the pedicles of the fractured vertebra with elevation. Once the cannula reached the center of the vertebral body, under continuous fluoroscopic monitoring, hydroxyapatite (HA) blocks (Olympus) were pushed into the defect to completely fill the anterior region of the defect for vertebral reconstruction. This was combined with fixation of the two vertebrae above and one below with a pedicle screw. Another 18 patients received balloon kyphoplasty (BKP), and 38 patients underwent anterior spinal decompression and fusion, performed using a titanium cage and supplemented with posterior percutaneous pedicle screw fixation (ADF + PF).

### Postoperative mortality

The mean follow-up time for survival was 4.1 ± 0.8 years. The primary endpoint was death by the close of the study period, which ended in March 2019. Data were censored if the patient survived until the end of the study period. If a recent follow-up was not available from clinical records, patients were contacted by telephone to confirm their survival.

### Data collection

For each patient who underwent spinal surgery for OVF, we searched clinical records and a laboratory database. Only measurements recorded 1–14 days before the date of surgery were used for preoperative measurements of body mass index (BMI), left ventricular ejection fraction (LVEF) measured using an echocardiogram, serum albumin, B-type natriuretic peptide (BNP), creatinine clearance (CCr), and preoperative neurological impairment (according to a modified Frankel classification). Standardized bone mineral density (BMD, % young adult men [YAM]) measurements at the lumbar spine (L2–L4) and the femoral neck were performed using a Lunar Prodigy dual-energy X-ray absorptiometry system (General Electric) at baseline. The American Society of Anesthesiologists (ASA) classification is a readily available and widely-accepted method for stratification of surgical patients according to their perioperative risk [13]. The visual analog scale (VAS) scores for the lumbar spine and leg were evaluated at the time of hospital admission and 1 year after surgery. Radiographic classification of burst fractures was based on the Denis classification using preoperative computed tomography [14].

### Statistical analyses

Continuous variables were compared using unpaired *t*-tests and categorical variables were assessed using Fisher’s exact tests for baseline characteristics between deceased patients and those who had survived for more than 2 years after surgery. Therefore, patients who had been followed for more than six months but less than two years were excluded in our analysis for Table 3. Crude and adjusted hazard ratios (HRs) of mortality, using Cox proportional univariate and multivariate models, were calculated. We also made Kaplan-Meier estimates of all-cause mortality according to serum albumin levels as a possible risk factor of death in the Cox multivariate model. A log-rank test was used to assess the significance of differences between Kaplan–Meier estimates. All statistical calculations were conducted using Prism software, version 8.0 (Graph Pad Software, La Jolla, CA) and SAS statistical software, version 9.4 (SAS Institute, Cary, NC, USA). R statistical software (version 3.6.1, R Project for Statistical Computing, Vienna, Austria) was used to generate Kaplan-Meier estimates. All reported *p*-values were two-sided and *p* < 0.05 indicated statistical significance.

## Results

### Patient characteristics between those who died during the follow-up period and those who survived 2 years after surgery

**Fig. 1:**
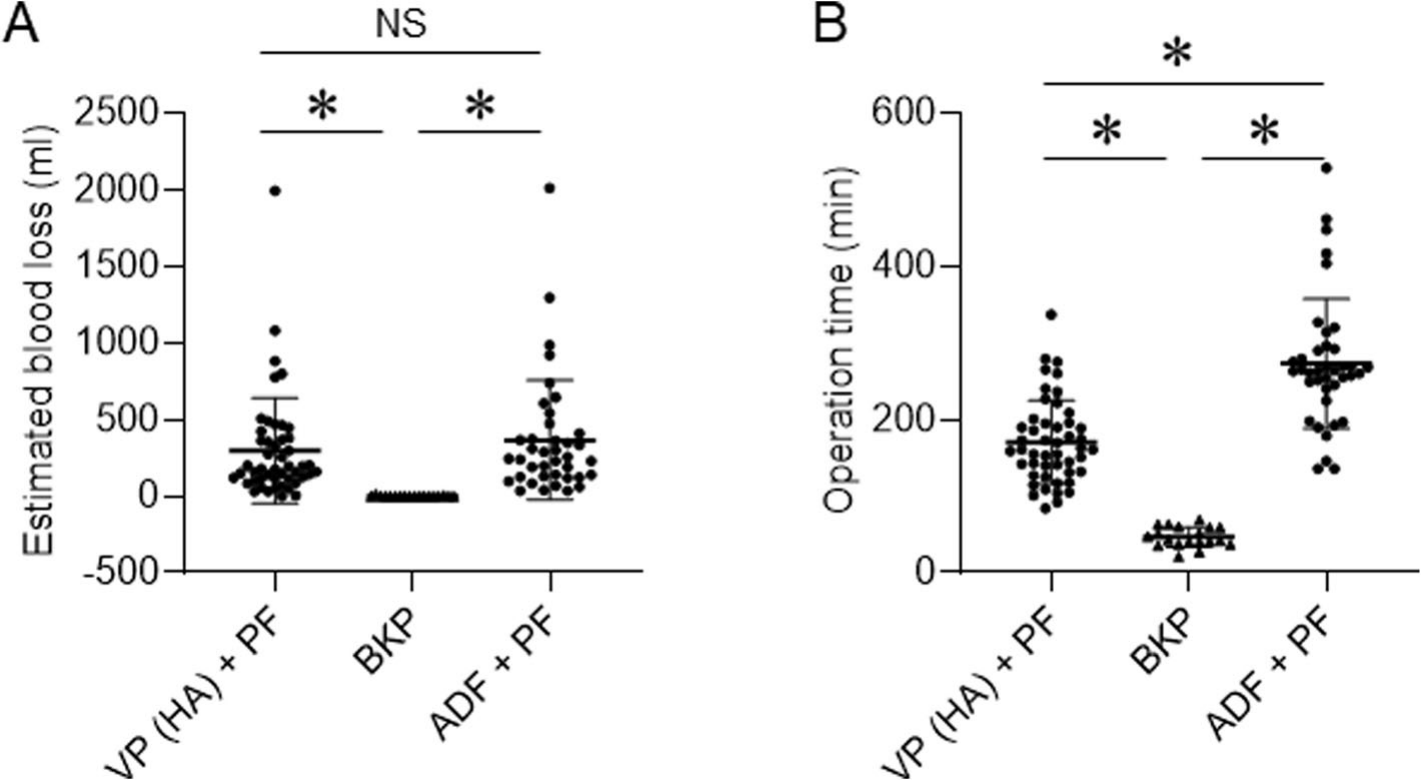
Comparisons of blood loss (**a**) and surgical time (**b**) among surgical procedures. ADF, anterior spinal decompression and fusion; BKP, balloon kyphoplasty; HA, hydroxyapatite; PF, posterior fixation; VP, vertebroplasty. *P < 0.05; NS = not significant. Data were analyzed using the unpaired t-test

Table 1 summarizes the characteristics of patients with OVF who underwent surgical procedures. Blood loss and surgical time for different surgical procedures were compared in Fig. 1. Estimated blood loss in patients treated with BKP was significantly lower than that in patients treated with VP (HA) + PF or ADF + PF. There was no significant difference in estimated blood loss between patients treated with VP (HA) + PF and ADF + PF (Fig. 1a). Surgical time in patients treated with BKP was significantly shorter than that in patients treated with VP (HA) + PF or ADF + PF. Surgical time in patients treated with VP (HA) + PF was significantly shorter than that in patients treated with ADF + PF (Fig. 1b). The VAS scores for both the lumbar spine and leg and modified Frankel classification were significantly improved one year after surgery compared to scores at the time of hospital admission (Table 2).

**Table 2:** Pre- and postoperative patient VAS scores (lumbar and leg) and Modified Frankel classification

	Preoperative	1 year after surgery	*p*-value
VAS score (lumbar)	7.4 ± 3.4	5.8 ± 3.8	< 0.05*
VAS score (leg)	6.7 ± 2.8	3.4 ± 2.1	< 0.05*
Modified Frankel classification (A/B/C/D/E)	0/0/44/39/22	0/0/28/42/31	< 0.05^†^

*Mean ± standard deviation, unless otherwise indicated

*P < 0.05 in the comparison between groups

^†^*p* value for Fisher’s exact test for Modified Frankel classification C versus D or E

*VAS* visual analog scale

Two years after surgery, 19/75 patients (15%) with OVF had died. Table 3 summarizes the preoperative baseline characteristics of the patients with OVF who died compared with those who were still alive two years after surgery. There were no significant differences in the age, BMI, LVEF (%), BNP level, CCr, or BMD, follow-up period, time from original injury to surgery, distribution of preoperative fracture type based on Denis’s classification, or estimated blood loss and operative time between groups. A significant difference in mortality among the three surgical procedures was not found (Table 3). In contrast, the ratio of males-to-females was significantly higher among the patients with OVF who died than for those who were still alive. Additionally, preoperative serum albumin levels and CCr were significantly lower in patients who died than in those who were still alive (Table 3).

**Table 3:** Factors associated with subsequent survival/mortality in patients who survived more than 2 years after surgery

Characteristic	Died (*n* = 19)	Survived (*n* = 56)	*p*-value^†^	Power
Male gender, n (%)	11 (57.9)	16 (28.6)	0.029*	0.48
Age, years	79.1 (7.6)	75.6 (8.0)	0.10	0.38
BMI, kg/m^2^	21.3 (3.1)	22.5 (3.0)	0.15	0.31
LVEF, %	70.3 (9.6)	71.9 (14.1)	0.65	0.078
Serum albumin, g/dL	3.4 (0.5)	3.9 (0.5)	0.0016*	0.96
BNP, pg/mL	45.3 (36.4)	44.9 (47.2)	0.98	0.050
CCr, mL/min	49.9 (20.1)	63.1 (21.6)	0.021*	0.65
BMD, %YAM	63.4 (14.0)	66.8 (13.4)	0.47	0.15
Follow-up, months	57.1 (21.2)	66.4 (25.3)	0.13	0.32
Time from original injury to surgery, months	4.4 (2.1)	4.6 (0.49)	0.21	0.090
Type of Denis’s classification (A/B.C)	12/7	34/21	0.72	
Surgical procedures			0.087	
VP (HA) + PF	11 (57.9)	27 (48.2)		
BKP	2 (10.5)	16 (28.6)		
ADF + PF	6 (31.6)	13 (23.2)		
ASA classification ≥3	7 (36.8)	6 (10.7)	0.016*	
Modified Frankel classification				
C versus D or E	8 (47.1)	21 (38.2)	0.58	
Estimated blood loss, ml	168 (155)	335 (499)	0.11	
Operative time, min	162 (98.9)	201 (114.3)	0.13	

^†^p value for Fisher’s exact test or Student’s t test

*P < 0.05 in the comparison between groups

Variables are presented as mean (standard deviation) unless noted otherwise

*ADF* anterior spinal decompression and fusion, *ASA* American Society of Anesthesiologists, *BMD* bone mass density, *BKP* balloon kyphoplasty, *n* number in group, *BMI* body mass index, *BNP* B-type natriuretic peptide, *CCr* creatinine clearance, *HA* hydroxyapatite, *LVEF* left ventricular ejection fraction, *PF* posterior fixation, *VP* vertebroplasty, *YAM* young adult male

### Preoperative factors affecting the long-term survival of the patients who underwent spinal surgery for an insufficient bone union following OVF

The presurgical hazard ratios (95% confidence interval) for mortality associated with patient characteristics are summarized in Table 4. According to a past report, hypoalbuminemia was defined as serum albumin ≤3.5 g/dL [15]. The univariate analysis showed that male gender, serum albumin < 3.5 g/dl, CCr < 60 mg/dl, and an ASA classificat0ion ≥3 were significant risk factors for postoperative mortality (Table 4). The multivariate analysis revealed that hypoalbuminemia was the only significant risk factor for long-term postoperative mortality of OVF (adjusted hazard ratio was 3.37; *p* = 0.014; Table 4).

**Table 4:** Pre-surgery hazard ratios (95% CI) of mortality based on patient characteristics (N = 105)

Explanatory variable	Crude	p-value	Adjusted HR^†^	p-value
Male vs female gender	2.74 (1.17, 6.41)	0.021	1.82 (0.76, 4.40)	0.18
BMI ≥25 vs 18.5–24.9 kg/m^2^ BMI ≥25 vs. 18.5–24.9 kg/m^2^	1.45 (0.41, 5.09)	0.56	1.67 (0.44, 6.32)	0.45
BMI < 18.5 vs 18.5–24.9 kg/m^2^ BMI < 18.5 vs. 18.5–24.9 kg/m^2^	2.78 (0.99, 7.81)	0.052	2.81 (0.96, 8.25)	0.061
Serum albumin < 3.5 g/dL	4.99 (2.00, 12.43)	0.0006	3.37 (1.28, 8.89)	0.014
CCr < 60 mg/dL	3.40 (1.15, 10.06)	0.027	1.96 (0.63, 6.05)	0.25
ASA physical status classification ≥3	3.93 (1.68, 9.21)	0.0017	2.36 (0.93, 6.01)	0.072

^†^Explanatory variables were selected using *p* < 0.2 in the univariate analysis for crude hazard ratios. All of the variance inflation factors in the multivariate model for the calculation of adjusted hazard ratios were < 1.22, indicating there was no multicollinearity

*ASA* American Society of Anesthesiologists, *BMI* body mass index, *CCr* creatinine clearance, *CI* confidence interval, *HR* hazard ratio

### Effect of preoperative serum albumin level on postoperative mortality

**Fig. 2:**
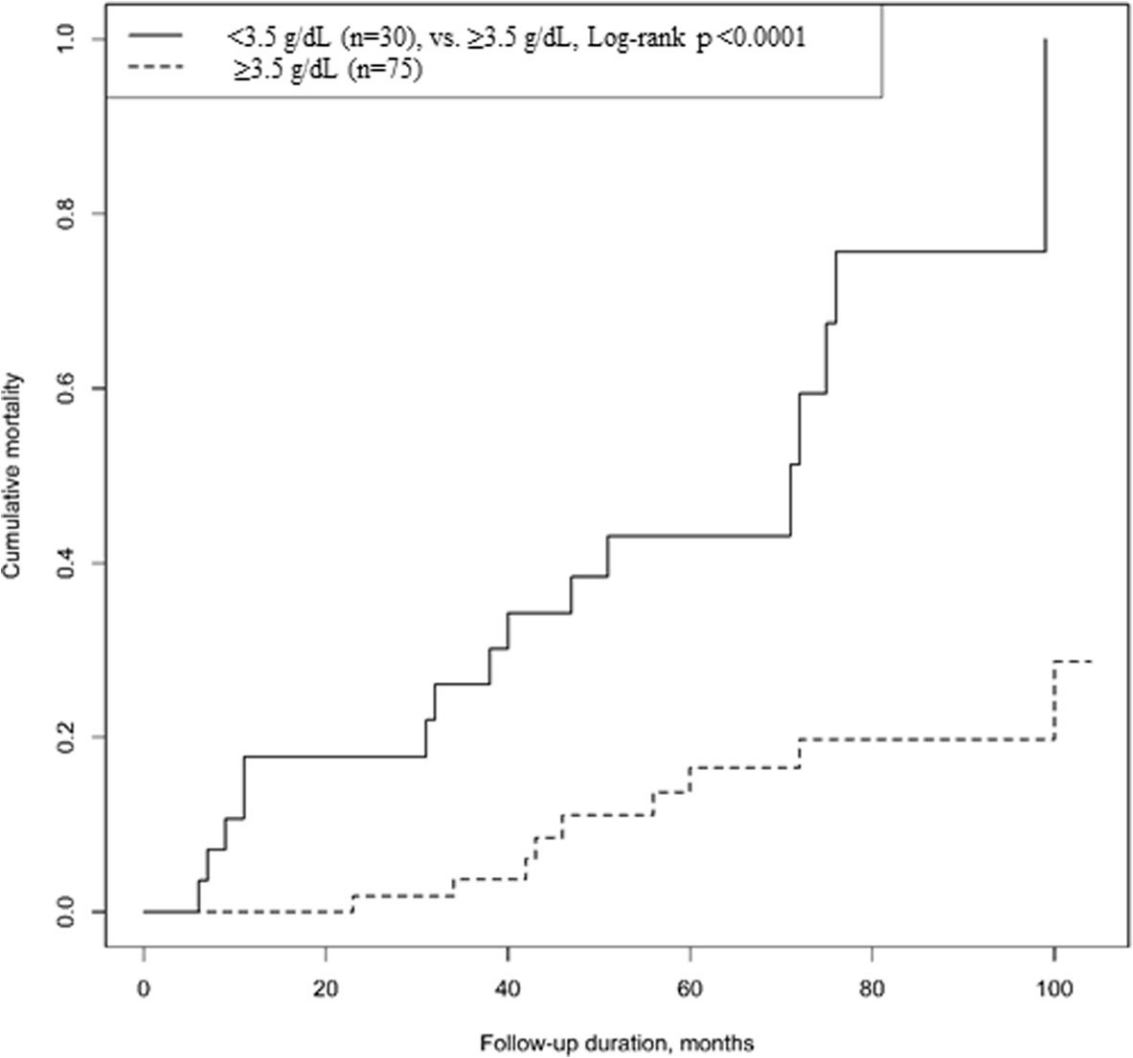
Overall survival comparison based on preoperative serum albumin levels (*dashed line*, ≤3.5 g/dL; *solid line*, > 3.5 g/dL) using the Kaplan-Meier method

Based on the threshold values defined, there were two preoperative serum albumin levels, ≤3.5 g/dL and > 3.5 g/dL [14]. Thirty-eight patients (36.2%) had hypoalbuminemia. The estimated risk of mortality at the final follow-up, using the Kaplan-Meier method, was significantly greater in patients with preoperative serum albumin ≤3.5 g/dL than in those with a serum albumin > 3.5 g/dL (*p* < 0.0001 for log-rank test; Fig. 2).

## Discussion

Numerous studies have found an association between OVF and increased mortality, with pulmonary and cardiovascular-related deaths suggested as being explanatory factors for the excess mortality [14–16]. A past study showed a 2- to 8-times increased risk of age-matched mortality following OVF [16]. Surgical intervention is often recommended for patients who have an insufficient bony union of OVF, persistent back pain, and/or neurological deficits [17]. Recently, interest has increased in the mortality and morbidity of patients who undergo spinal surgery [18, 19]. Additionally, numerous reports have surfaced for comparative studies of surgical procedures for OVF; these have focused on surgical invasiveness and complications [20]. By contrast, the postoperative outcome of pain and survival of patients with an insufficient bony union after OVF surgery remains largely unknown. Although the present study showed that the VAS scores for both the lumbar spine and leg were significantly improved one year after surgery compared to scores at the time of hospital admission, those scores may not be meaningful clinically. A recent study indicated that instrumented fusion surgery for lumbar trauma in the elderly is associated not only with increased morbidity, but also with reduced mortality [21]. Therefore, we sought to elucidate the preoperative risk factors which may predict postoperative mortality. Interestingly, our study indicated no significant differences in postoperative mortality among the 3 different surgical procedures assessed for OVF.

The association between malnutrition and mortality risk has been shown in patients with hip fractures [22, 23]. Additionally, a recent study indicated that serum albumin levels predict which patients are at increased risk for minor or major complications, or mortality, following surgical management of acute osteoporotic vertebral compression fractures [24]. We presumed that patients with long-term post-injury OVF may be at increased risk of malnutrition due to ongoing pain compared to acute fractures. Serum albumin is a well-known and important marker of preoperative nutritional status. Low levels of serum albumin can be prognostic by influencing organ vascularization and may hinder antibiotic therapy, prolong inflammation, and prompt intravascular coagulation [25]. Numerous oncology studies have established a preoperative serum albumin level of ≤3.5 g/dL as the cut-off value for risk of poor overall survival [26, 27].

According to past studies, preoperative serum albumin levels were significantly lower in the patients with OVF who died, than in those who remained alive. The present study also showed a significant association between a low preoperative serum albumin level of ≤3.5 g/dL, and a reduction in overall survival for patients undergoing surgery for an insufficient union of OVF.

This study has some limitations. First, this study was retrospective and had a small sample size and duration of follow-up; a prospective study and a longer follow-up time would have been preferable. Second, we do not know whether surgical therapy or a difference in surgical techniques had an impact on the patients’ prognosis because an individual spine surgeon might decide on surgery depending on the patient’s general condition. Further study is needed to clarify the strategy for selecting surgical procedures, and these studies should include OVF patients treated with conservative therapy. Third, the differences between patients with injuries at different spinal levels should be investigated with a larger sample size. However, the present study clearly shows that hypoalbuminemia is particularly important among the many predicted risk factors for increased postoperative mortality following OVF surgery. This result suggests that designing a treatment strategy that takes into consideration indications and optimal timing for OVF surgery may have clinical significance. Based on the present study, we propose that if OVF patients are found to have poor nutrition by preoperative examination, attention to nutrition should be paid postoperatively, and/or less invasive procedures may be considered. Additionally, efforts should be made to improve nutritional status before surgery. Further studies are needed to investigate the effects on postoperative mortality of improving nutritional status before surgery.

## Conclusion

Hypoalbuminemia was associated with increased postoperative mortality following OVF surgery.

## Data Availability

The datasets used and/or analyzed during the current study are available from the corresponding author on reasonable request.

## Abbreviations

OVF: Osteoporotic vertebral fractures
LSS: Lumbar spinal canal stenosis
BMI: Body mass index
LVEF: Left ventricular ejection fraction
BNP: B-type natriuretic peptide
CCr: level, Creatinine clearance
BMD: Bone mass density
ASA: The American Society of Anesthesiologists
